# Human Norovirus Evolution in a Chronically Infected Host

**DOI:** 10.1128/mSphere.00352-16

**Published:** 2017-03-29

**Authors:** Sylvie Y. Doerflinger, Stefan Weichert, Anna Koromyslova, Martin Chan, Christian Schwerk, Ruediger Adam, Stefan Jennewein, Grant S. Hansman, Horst Schroten

**Affiliations:** a Department of Infectious Diseases, Virology, University of Heidelberg, Heidelberg, Germany; b Schaller Research Group at the University of Heidelberg and the DKFZ, Heidelberg, Germany; c Pediatric Infectious Diseases Unit, University Children’s Hospital Mannheim, University of Heidelberg, Mannheim, Germany; d Department of Microbiology, Faculty of Medicine, The Chinese University of Hong Kong, Hong Kong, China; e Jennewein Biotechnology, Rheinbreitbach, Germany; University of Pittsburgh School of Medicine

**Keywords:** chronic infection, evolution, norovirus

## Abstract

Typically, human noroviruses cause symptoms of acute gastroenteritis for 2 to 4 days. Often, the virions are shed in stool for several days after the symptoms recede, which in turn can lead to further contamination and transmission. Moreover, a number of reports have considered that chronic norovirus infections, i.e., lasting months and years, might even function as reservoirs for the generation of novel strains that can escape the herd immunity or have modified binding interactions with histo-blood group antigens (HBGAs). In this study, we analyzed noroviruses isolated from a patient who has presented a chronic infection for more than 6 years. We found that the isolated capsid sequences clustered into two main genetic types (termed A and B), despite a plethora of capsid quasi-sequences. Furthermore, the two genetic types corresponded well with distinct antigenicities. On the other hand, we showed that numerous amino acid substitutions on the capsid surface of genetic types A and B did not alter the HBGA binding profiles. However, divergent binding profiles for types A and B were observed with human milk oligosaccharides (HMOs), which structurally mimic HBGAs and may act as natural antivirals. Importantly, the isolated capsid sequences only had approximately 90% amino acid identity with other known sequences, which suggested that transmission of these chronic noroviruses could be limited.

**IMPORTANCE** The norovirus genogroup II genotype 4 (GII.4) variants have approximately 5% divergence in capsid amino acid identity and have dominated over the past decade. The precise reason(s) for the GII.4 emergence and persistence in the human population is still unknown, but some studies have suggested that chronically infected patients might generate novel variants that can cause new epidemics. We examined GII.4 noroviruses isolated from an immunocompromised patient with a long-term infection. Numerous norovirus capsid quasi-species were isolated during the 13-month study. The capsid quasi-species clustered into two genetic and antigenic types. However, the HBGA binding profiles were similar between the two antigenic clusters, indicating that the amino acid substitutions did not alter the HBGA binding interactions. The isolated sequences represented two new GII.4 variants, but similar sequences were not found in the database. These results indicated that chronically infected patients might not generate novel noroviruses that cause outbreaks.

## Abstract (precis)

The norovirus genogroup II genotype 4 (GII.4) variants have approximately 5% divergence in capsid amino acid identity and have dominated over the past decade. The precise reason(s) for the GII.4 emergence and persistence in the human population is still unknown, but some studies have suggested that chronically infected patients might generate novel variants that can cause new epidemics. We examined GII.4 noroviruses isolated from an immunocompromised patient with a long-term infection. Numerous norovirus capsid quasi-species were isolated during the 13-month study. The capsid quasi-species clustered into two genetic and antigenic types. However, the HBGA binding profiles were similar between the two antigenic clusters, indicating that the amino acid substitutions did not alter the HBGA binding interactions. The isolated sequences represented two new GII.4 variants, but similar sequences were not found in the database. These results indicated that chronically infected patients might not generate novel noroviruses that cause outbreaks.

## INTRODUCTION

Human noroviruses are the dominant cause of outbreaks of acute gastroenteritis. The disease is typically self-limiting, with most symptoms lasting two to four days. However, noroviruses can be shed in stool for several weeks after an infection and asymptomatic individuals can transmit infectious particles to other individuals, which can also lead to additional outbreaks (1). In general, norovirus person-to-person transmission is associated with one strain, whereas food-borne outbreaks can comprise more than one variant (2, 3). Immunity to human norovirus is still unknown, meaning that previous infections might not provide protection against additional norovirus exposure. Indeed, human trials have indicated that norovirus vaccine candidates may only reduce the severity of the disease (4), while long-term protection remains uncertain (5). Thus, human noroviruses are a major burden in the community.

Human noroviruses are genetically and antigenically diverse (6), yet a single genetic cluster (genogroup II genotype 4 [GII.4]) has dominated over the past decade (7). A number of studies have shown that ~5% of the GII.4 noroviruses evolve into new genetic variants every year, and they are believed to have a mechanism that allows the virus to evade the immune system or alter receptor binding profiles (8–10). Moreover, genetic recombination, which is not uncommon within the GII.4 genotype, increases their diversity (11).

Several reports have identified chronic norovirus infections lasting months to years, especially in immunocompromised individuals (12–16). Some studies have speculated that these chronically infected individuals might even function as reservoirs for the generation of novel noroviruses that could be transmitted into the community and escape the herd immunity (17, 18). On the other hand, a recent molecular epidemiological analysis of chronically infected patients found no evidence for this kind of transmission (19). Therefore, the idea of a reservoir(s) for novel strains and the transmissibility of chronic noroviruses are still controversial.

The norovirus genome is divided into three open reading frames (ORFs), where ORF1 encodes the nonstructural proteins, including the RNA-dependent RNA polymerase (RdRp), ORF2 encodes the capsid protein (VP1), and ORF3 encodes a small structural protein. The expression of the norovirus capsid protein in insect cells leads to the self-assembly of virus-like particles (VLPs) that are morphologically similar to the native virion (6). The norovirus capsid is composed of two main domains, the shell (S) and protruding (P) domains (20). The S domain forms a protective scaffold for the RNA, whereas the P domain contains the main determinants of antigenic diversity.

The norovirus capsid interacts with host histo-blood group antigens (HBGAs). This interaction with HBGAs is known to be important for virus entry and replication (21, 22), and genetic polymorphism in the genes that control HBGA synthesis provides an intraspecies diversity. In addition, one study suggested that norovirus HBGA binding profiles might change over time in chronically infected patients (23).

Despite their discovery more than four decades ago, there are still no approved vaccines or antivirals available for human noroviruses. However, human milk oligosaccharides (HMOs), which mimic HBGA structures, were recently shown to block norovirus VLPs from binding to HBGAs (24), making them suitable candidates to possibly control human norovirus infection.

In this study, we analyzed noroviruses isolated from an immunocompromised patient presenting a long-term infection in order to examine the hypothesis that chronically infected individuals are reservoirs for clinically relevant noroviruses. We identified a plethora of novel capsid quasi-species that showed varied cross-reactivities and binding interactions with HBGAs and HMOs. Importantly, similar capsid sequences are not yet reported, suggesting that these viruses were not easily transmitted to other individuals.

## RESULTS

### Analysis of the capsid gene.

A total of 186 full-length capsid genes from the patient were isolated and sequenced. All sequences belonged to GII.4. Four of 186 sequences contained premature stop codons near the C terminus, while 182 were of the putative full-length size. Interestingly, an earlier study showed that norovirus capsids with C-terminal deletions produced VLPs with larger diameters but reduced stability compared to those of native-size particles (25). Therefore, these results suggested that the norovirus in this chronic patient might have produced modified capsids.

**FIG 1:**
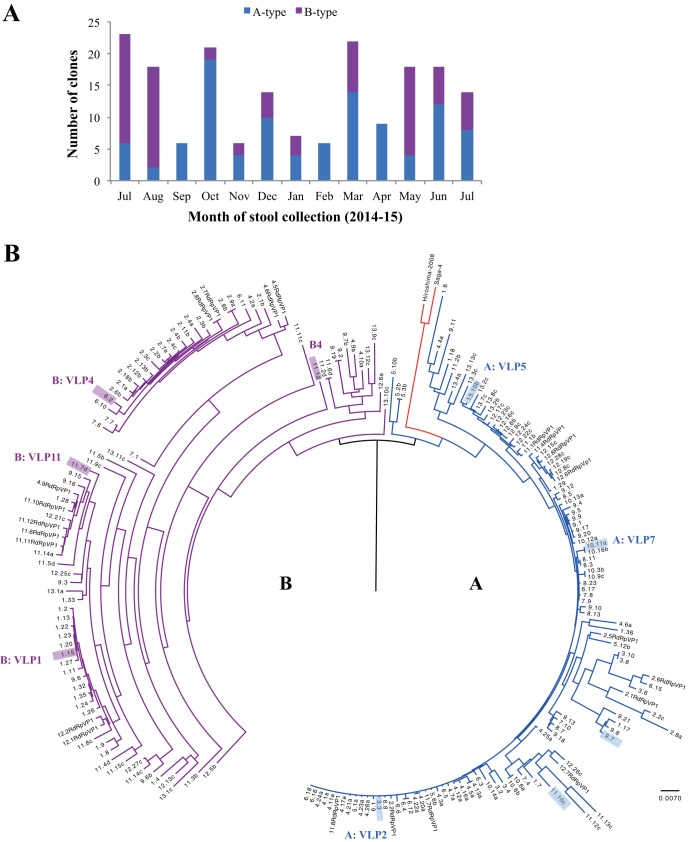
Analysis of isolated norovirus capsid quasi-sequences (labeled month.clone, e.g., 4.21a refers to month 4 and clone 21a). (A) Distribution of the type A and B capsid sequences. The numbers of capsid clones sequenced in each month were 23 (month 1), 18 (month 2), 6 (month 3), 21 (month 4), 6 (month 5), 14 (month 6), 7 (month 7), 6 (month 8), 22 (month 9), 9 (month 10), 19 (month 11), 18 (month 12), and 14 (month 13). (B) Nucleotide capsid sequences (182 capsid clones and 22 capsids from RdRp-VP1) with the gaps removed were aligned using ClustalW. The sequences were divided into types A (blue) and B (purple), along with reference sequences from Saga-4 (GenBank AB447457) and Hiroshima-2008 (GenBank AB541252). The scale bar represents the number of nucleotide substitutions per position. VLPs were produced for types A (VLP2, VLP5, and VLP7: clones 3.3, 13.10b, and 10.11a, respectively) and B (VLP1, VLP4, and VLP11: clones 1.15, 6.2, and 11.7d, respectively). See Fig. 5A and B for P domain homology models for sequences of type A (sequences 3.3, 9.7, 11.10c, and 13.10 b [shaded blue]) and type B (sequences 1.15, 6.2, 11.7d, and 11.1d [shaded purple]).

Genetic analysis showed that a plethora of capsid quasi-sequences were generated over the 13-month period. However, the 182 capsid sequences clustered into two main genetic types (termed A and B), which encoded 540 and 539 amino acids, respectively (Fig. 1). The nucleotide sequence similarities between types A and B were 90 to 100%. This difference was typical for an intragenotype variation (26, 27), although types A and B could be considered genetically distinct, i.e., GII.4 variants. Of importance, the type B capsid sequences (539 amino acids) have typically been found prior to 2000, whereas the type A capsid sequences (540 amino acids) mainly circulated after 2000 and continue to cause the majority of outbreaks (27, 28).

**FIG 2:**
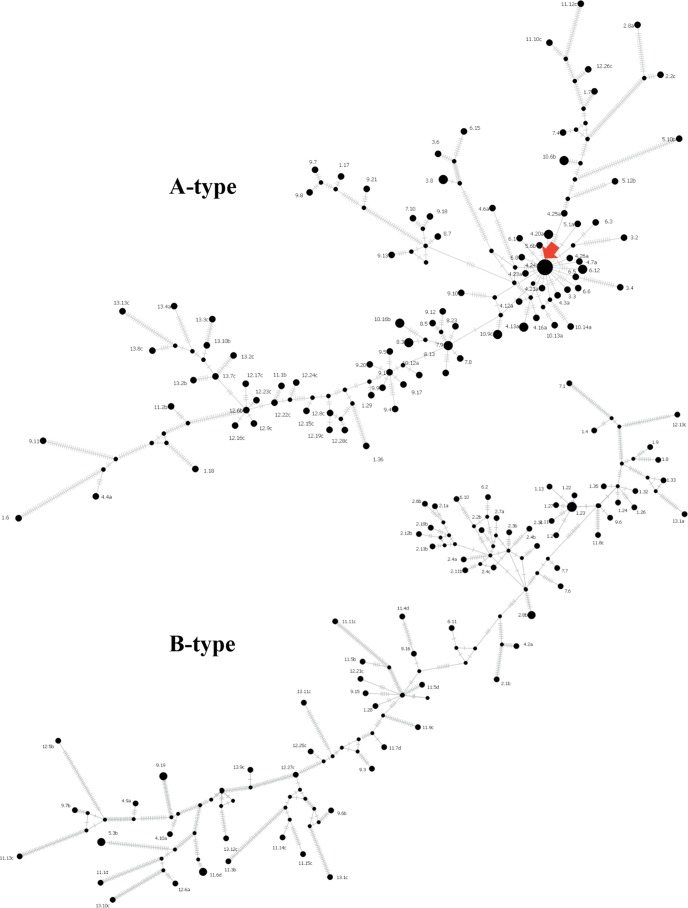
Median-joining haplotype network of 104 type A (top) and 78 type B (bottom) full-length capsid nucleotide sequences. Terminal circles represent sampled haplotypes with sizes proportional to the number of sequences. Haplotypes are labeled by the time in months since the collection of the first sample, followed by the clone number. Internal black dots indicate unsampled hypothetical haplotypes. The red arrow indicates a highly connected haplotype. Each hashed line denotes the number of nucleotide difference between the connected haplotypes. Connecting lines are not drawn to scale.

Interestingly, type A sequences were isolated in all months, whereas type B sequences were only detected in 9 months (Fig. 1A). Haplotype network analysis revealed the presence of a highly connected haplotype with the highest number of sampled capsid sequences in type A (Fig. 2). This may represent a competent haplotype, and the nonidentical topology may imply that types A and B evolved differently within the host.

**FIG 3:**
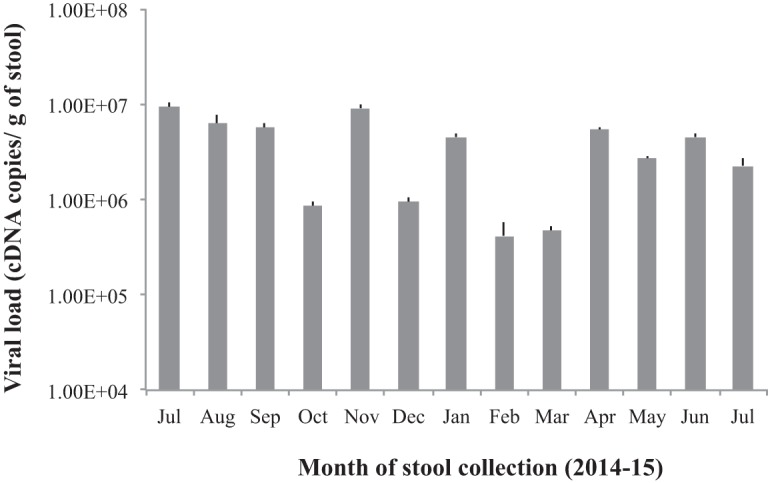
The viral load in each month. The viral load in each month was determined using quantitative real-time (RT)-PCR with a sense primer (5-ACDATYTCATCATCACCATA-3) and an antisense primer (5-TGGAATTCCATCGCCCACTGG-3). The viral loads are the mean results of duplicate runs, and the error bars show standard deviations.

The viral loads in each month ranged between 4.2 × 10^5^ and 9.4 × 10^6^ virus cDNA copies/g of stool (Fig. 3), which is comparable to the levels in acute infections (29). Human norovirus is highly contagious, with only 10 to 20 particles required for an infection (30). However, both parents of the chronic patient were found to be norovirus negative and had no reported episodes of norovirus infection during the 6 years of the patients’ chronic infection. This result could imply that the parents were previously infected and had acquired immunity against these strains. On the other hand, the closest matching sequences in the GenBank database had 90 to 91% nucleotide similarity with types A and B, which further suggested that these chronic viruses had limited transmission into the community.

**FIG 4:**
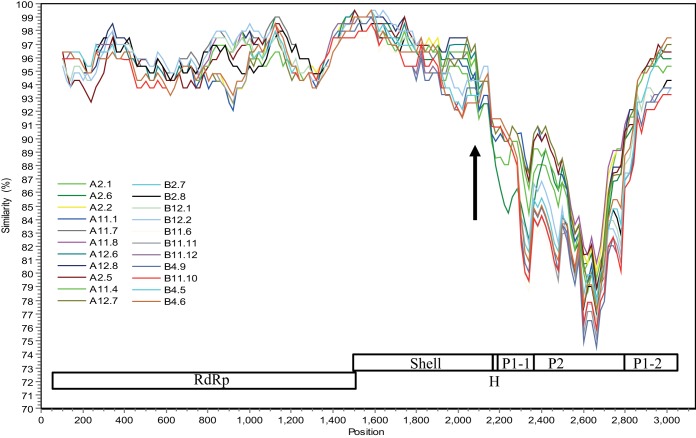
Recombination with the capsid gene. SimPlot analysis of 22 cloned RdRp-capsid nucleotide sequences (start of RdRp to end of capsid) with Saga-4 as a reference, using a window size of 200 bp and increments of 20 bp with all gaps removed. The schematics of the RdRp (nt 1 to 1531), shell domain (nt 1514 to 2170), hinge region (H, nt 2171 to 2186), P1-1 subdomain (nt 2187 to 2350), P2 subdomain (nt 2351 to 2760), and P1-2 subdomain (nt 2761 to 3130) are shown. The arrow represents the recombination breakpoint.

In order to identify genetic recombination at the definitive RdRp and capsid junction recombination hot spot (11), a single reverse transcription (RT)-PCR fragment covering the RdRp-to-capsid (RdRp-capsid) genes from representative months 2, 4, 11, and 12 was sequenced (22 sequences in total). The RdRp and capsid sequences both belonged to GII.4. The capsid sequences clustered into genetic types A and B (Fig. 1B). The RdRp sequences closely matched that of the Saga-4 strain (identified in 2006), having ~99% amino acid identity, whereas the capsid sequences had 90% amino acid identity. A SimPlot analysis of the 22 sequences showed a sudden drop in nucleotide similarity immediately after the S domain and then an increase after the P2 subdomain (Fig. 4). This result suggested a possible recombination event, where the P1-1/P2 domains were replaced, although the P1-2 subdomain may have also been replaced. Generally, the P2 subdomain is the most variable region and is considered an insert in the capsid (20). Indeed, recombination events at the S and P1-1 domains were also observed for other GII.4 variants (11).

### Homology model of P domains.

**FIG 5:**
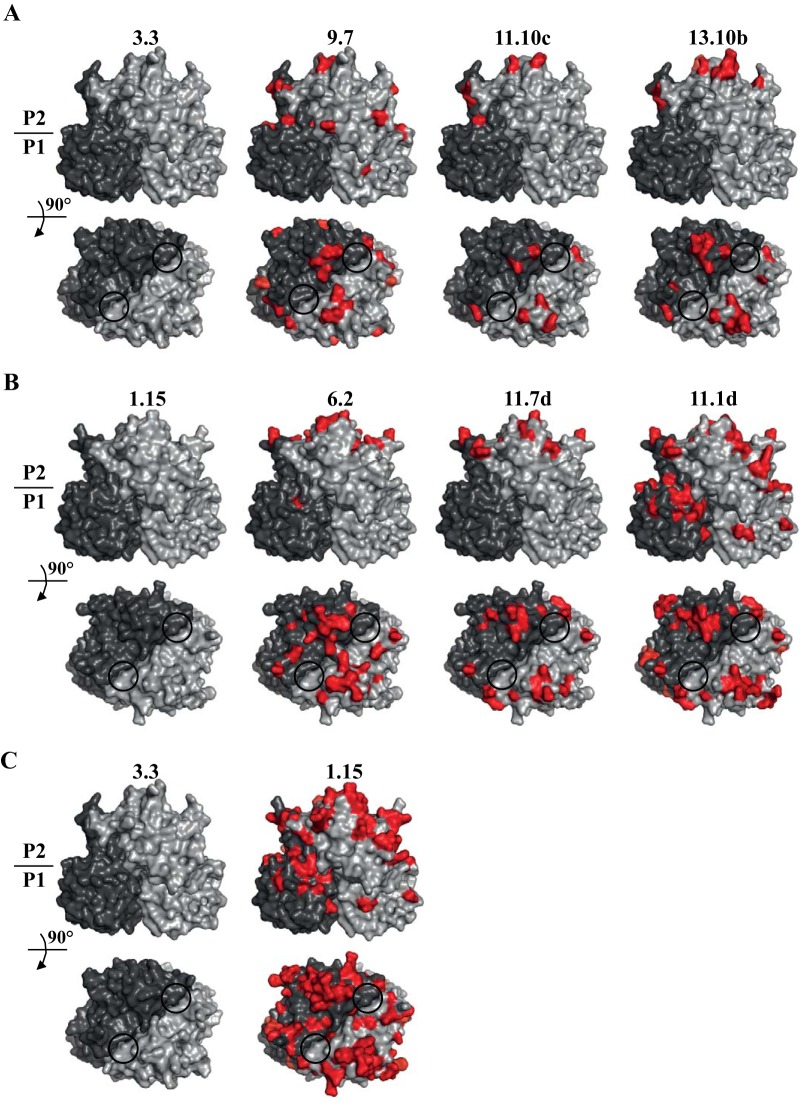
Amino acid substitutions on the P domains. (A and B) P domain homology models were created for different clusters (see Fig 1A and 2) in type A (sequences 3.3, 9.7, 11.10c, and 13.10 b) (A) and type B (sequences 1.15, 6.2, 11.7d, and 11.1d) (B). These variant sequences represent different branches based on the phylogenetic analysis (see Fig. 1B). The different grays represent each monomer, red shows the amino acid substitutions, and the black circles indicate the HBGA binding pockets. (C) Amino acid differences between type A (sequence 3.3) and type B (sequence 1.15). Substantial variations were observed throughout the P domain, whereas the region below the HBGA pocket remained relatively unchanged.

The amino acid differences among types A (sequences 3.3, 9.7, 11.10c, and 13.10 b) and B (sequences 1.15, 6.2, 11.7d, and 11.1d) were plotted onto P domain homology models in order to illustrate sequence variations (Fig. 5). For type A, most amino acid substitutions were located on the P2 subdomain and on surface-exposed loops (Fig. 5A). The residues below the HBGA pockets remained relatively unchanged, whereas the surrounding regions showed a number of substitutions. For type B, amino acid substitutions appeared on the P2 subdomain and were also located on the P1 subdomain (Fig. 5B). Similar to the A type, the region below the B type HBGA pocket remained relatively unchanged. Comparing the A and B types, there were notable amino acid substitutions in both the P1 and P2 subdomains, although the region directly below the HBGA pocket remained unchanged (Fig. 5C). Previous studies indicated that saccharide residues other than the fucose moiety were held with various residues and water-mediated interactions (31–34). Therefore, substitutions outside the fucose-binding pocket could be tolerated and still bind HBGAs. Interestingly, four of five residues (Asp374, Arg345, Thr344, Tyr444, and Gly443) that regularly interact with the fucose moiety of the HBGAs remained unchanged, except for Tyr444, which was a histidine (His444) in all of the capsid sequences isolated. Structural analysis of other epidemic GII.4 P domains indicated that an equivalent His444 residue was absent. This result suggested that these chronic norovirus capsids might interact with HBGAs differently than the epidemic GII.4 noroviruses.

### Antigenic analysis of norovirus VLPs.

**FIG 6:**
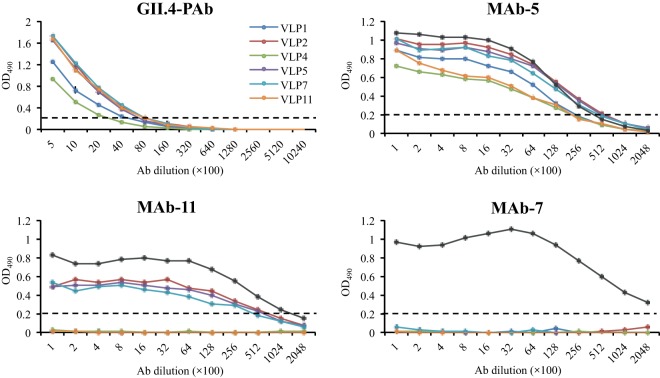
Cross-reactivities of VLPs. The VLPs were normalized to the same starting concentrations. Samples were analyzed in duplicates; error bars show standard deviations (see also Fig. S1 in the supplemental material). The cutoff (OD_490_ of 0.2) is indicated with dashed lines. The GII.4 PAb cross-reacted with all six VLPs. Four MAbs (1, 3, 5, and 6) cross-reacted with all VLPs. Three MAbs (4, 9, and 11) cross-reacted with only type A VLPs. Four MAbs (2, 7, 8, and 10) had no cross-reactivity with any of the VLPs. Saga-1 VLPs (GenBank AB447456; amino acid sequence identical to that of Saga-4) were used as a positive control in all MAb experiments (black line).

**FIG 7:**
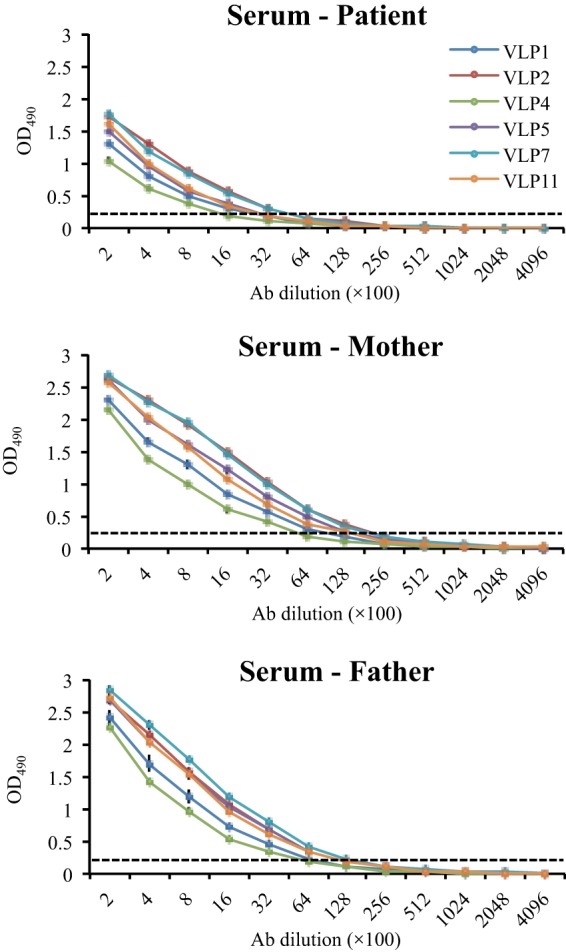
Cross-reactivities of the six VLPs against sera from the patient and his parents. The cutoff (OD_490_ of 0.2) is shown with dashed lines. All VLPs reacted with the human sera. The parents’ antibody titers were approximately 4-fold higher than that of the patient. Experiments were performed in duplicates; the error bars show standard deviations.

In order to better describe the capsid phenotypes, the antigenic variations were analyzed with norovirus-specific antisera (11 monoclonal antibodies [MAbs] and 1 polyclonal antibody [PAb]) and six different VLPs that represented genetic types A and B (Fig. 6; see also Fig. S1 in the supplemental material). Four of the 11 MAbs were unreactive with the six VLPs but cross-reacted with the positive control (i.e., 2006 GII.4 Saga-1 VLPs). Three MAbs cross-reacted with only type A VLPs, while four MAbs and the PAb reacted with all of the VLPs. Taken together, these results indicated that the genetic types A and B also represented two distinct antigenic types. Interestingly, the patient’s and parents’ sera were all reactive to the type A and B VLPs (Fig. 7). These results further suggested that the parents had probably been infected with the same or antigenically similar noroviruses at some point in time.

10.1128/mSphere.00352-16.1 FIG S1  Cross-reactivities of the six VLPs against GII.4 antisera. The VLPs were normalized to the same starting concentration. The cutoff (OD_490_ of 0.2) is shown with dashed lines. Saga-1 VLPs (GenBank AB447456; these have an amino acid sequence identical to that of Saga-4) were used as a positive control in all experiments (black line). MAb-1, MAb-3, and MAb-6 cross-reacted with all VLPs. MAb-4 and MAb-9 cross-reacted with type A VLPs, i.e., VLP2, VLP5, and VLP7. MAb-2, MAb-8, and MAb-10 did not cross-react with any of the VLPs except the positive control. Download FIG S1, PDF file, 0.2 MB. Copyright © 2017 Doerflinger et al. 2017 Doerflinger et al. This content is distributed under the terms of the Creative Commons Attribution 4.0 International license.

### HBGA binding interactions.

**FIG 8:**
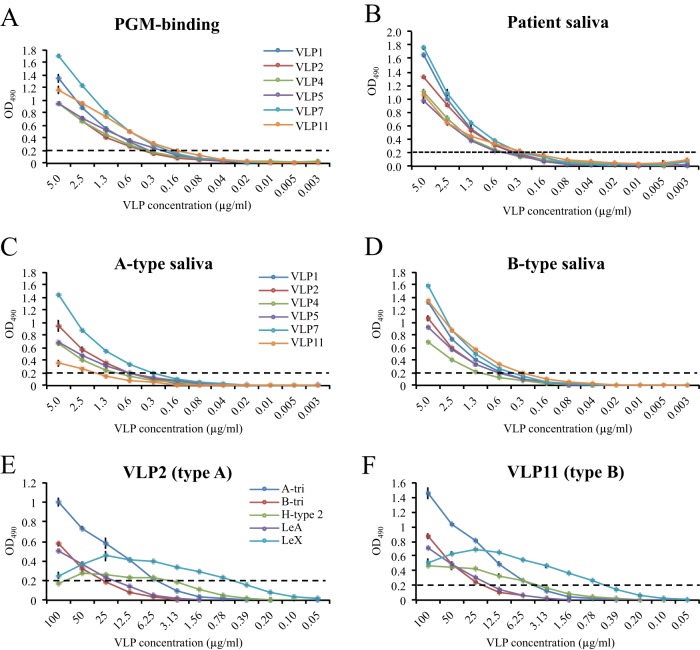
VLP binding interactions with HBGAs and saliva samples. The cutoff (OD_490_ of 0.2) is shown with dashed lines. Type A and B VLPs were serially diluted and added to duplicate wells. Samples were done in duplicates; error bars show standard deviations. (A) PGM, which contains a mixture of A and H types, was coated on ELISA plates. All of the VLPs were capable of binding to PGM in a dose-dependent manner. (B) VLP binding to patient’s saliva samples. Type A and B VLPs bound to the saliva with similar cutoff dilutions. (C and D) Type A and B VLPs were capable of binding to A- and B-type saliva. (E and F) VLPs representing types A (VLP2) and B (VLP11) were analyzed for their binding interactions with synthetic HBGAs (A trisaccharide, B trisaccharide, H2 trisaccharide, LeA trisaccharide, and LeX trisaccharide) using a slightly modified ELISA (47). The type A and B VLPs bound to the synthetic HBGAs with similar binding profiles.

The VLP binding interactions with HBGAs were determined using porcine gastric mucin type III (PGM), saliva samples, and synthetic HBGAs. We found that the type A and B VLPs bound to PGM at similar cutoff levels (i.e., 0.3 to 0.6 μg/ml) and with similar binding profiles (Fig. 8). All VLPs bound both A- and B-type saliva samples, as well as the patient’s saliva (Fig. 8B, C, and D). The VLPs also bound to several synthetic HBGAs with similar binding profiles for both type A and B VLPs (Fig. 8E and F). However, the binding cutoff limits for B trisaccharide and Lewis A (LeA) trisaccharide were lower than for A trisaccharide, H type 2 (H2) trisaccharide, and Lewis X (LeX) trisaccharide. One explanation for the low levels of binding to synthetic B trisaccharide and higher levels of binding to B-type saliva is likely the fact that B-type saliva contains H-type saccharides (35).

### HMO inhibition analysis.

**FIG 9:**
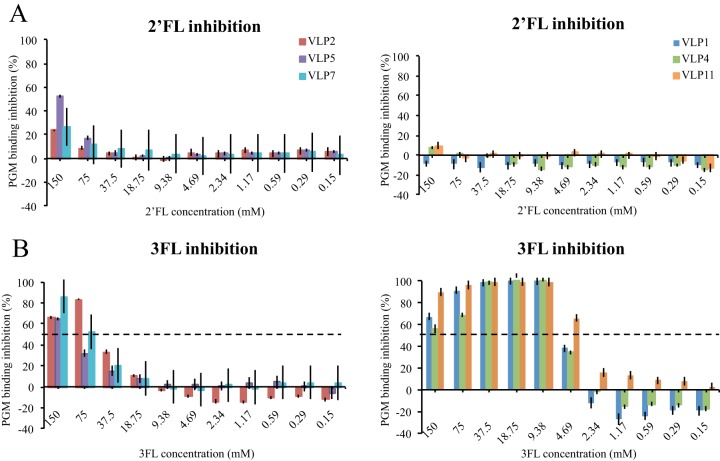
HMO blocking of VLP binding to HBGAs. HMO inhibition assay of VLPs binding to HBGAs. (A) 2′FL showed little or no inhibition of VLP binding to PGM, and the IC_50_s were not calculated. (B) 3FL showed dose-dependent inhibition. For type A VLPs (VLP2, VLP5, and VLP7), the 3FL IC_50_s were 60 mM, 116 mM, and 82 mM, respectively, while for type B VLPs (VLP1, VLP4, and VLP11), the IC_50_s were 6 mM, 6 mM, and 3 mM, respectively. Error bars show standard deviations.

Our previous data indicated that the HMOs 2′-fucosyllactose (2′FL) and 3FL inhibited GII.10 VLPs from binding to HBGAs (24). In order to compare the differences between type A and B VLPs, the inhibition was analyzed with an identical method (Fig. 9). 2′FL showed little inhibition of the binding of type A and B VLPs to PGM. For 3FL, a dose-dependent inhibition pattern was observed. The 3FL 50% inhibitory concentrations (IC_50_s) were 60 mM, 116 mM, and 82 mM for type A VLPs (VLP2, VLP5, and VLP7, respectively). For type B VLPs (VLP1, VLP4, and VLP11), the inhibition was considerably stronger (~10-fold), such that the 3FL IC_50_s were 6 mM, 6 mM, and 3 mM, respectively. Overall, these data showed that 3FL interacted differently with type A and B VLPs. Moreover, these results showed that 3FL’s inhibition of the binding of chronic and GII.10 VLPs was equivalent, whereas 2′FL’s inhibition of the binding of chronic VLPs was noticeably different than its inhibition of the binding of GII.10 VLPs (24).

## DISCUSSION

The main aim of this study was to investigate whether a chronically infected individual generated novel norovirus variants that were further transmitted into the community. For this purpose, we analyzed norovirus variants in an immunocompromised patient with long-term infection. We isolated a plethora of GII.4 capsid quasi-species during the 13-month period. The sequences clustered into two main genetic types (A and B) that also corresponded with two antigenic types. We found that the capsid sequences isolated had approximately 90% amino acid identity to other known sequences in the database. Therefore, these capsid sequences were assumed to represent novel GII.4 variants that have not yet been reported for any outbreaks.

Interestingly, both the patient and his parents exhibited antibodies that were reactive against the chronic noroviruses. Remarkably, the parents had not presented a norovirus infection during the study period, and yet, the viral loads in the chronic patient were equivalent to those found in acute infections. On the other hand, a recent study has also shown that chronically infected patients had not transmitted noroviruses to other individuals (19). These results suggested that chronic noroviruses might not maintain a pathogenesis similar to that of other GII.4 noroviruses circulating in the community, as suggested with other viruses (36). Still, the HBGA binding interactions of the chronic norovirus types A and B were similar to those of other noroviruses that caused acute infections.

Numerous molecular epidemiological studies have indicated that the GII.4 noroviruses evolve into new genetic variants every other year, leading to six major pandemic outbreaks since 1996 (37–41). We previously showed that several of these GII.4 variants were capable of binding numerous HBGA types (32). One study even suggested that the HBGA binding profiles might change over time in chronically infected patients (23). We showed that the two genetic types (A and B) isolated in this current study bound HBGAs in a similar manner, despite having surface-exposed substitutions adjacent to the HBGA pocket and approximately 10% amino acid divergence. Moreover, the HBGA binding interactions were comparable over the different representative months. Our results indicated that the viruses produced in the immunocompromised host had no selective pressure to alter HBGA binding interactions but, rather, produced antigenic variants that could bind HBGAs. In contrast, in other noroviruses that cause acute infections, in particular the recently emerging GII.17 noroviruses, both the antigenicity and the HBGA pocket appeared to have been altered (42, 43). Interestingly, four of five residues (Asp374, Arg345, Thr344, Tyr444, and Gly443) that regularly interact with HBGAs were maintained in the chronic norovirus sequences. One of the conserved residues (Tyr444) was replaced with a histidine (His444), which typically provided a hydrophobic interaction for GII.4 and GII.10 noroviruses (32, 33). However, the GII.12 noroviruses contained neither tyrosine nor histidine (33), which suggested that this residue could be replaced in certain noroviruses. Therefore, it is tempting to speculate that His444 was a signature residue in these chronic norovirus sequences. Further X-ray crystal structures of these P domain-HBGA complexes may help to determine the function of His444.

As mentioned above, our enzyme-linked immunosorbent assay (ELISA) data showed that the type A and B VLPs interacted with HBGAs in a similar manner. On the other hand, we found that 3FL’s inhibition of binding was different with types A and B, while 2′FL showed little inhibition with both type A and B VLPs. This result suggested that amino acid substitutions located near the HBGA pocket altered specific HMO binding interactions. We previously showed that 2′FL and 3FL bound to GII.10 P domains with various residues and different orientations, although the fucose moiety was held similarly (24). Although these HMOs have not yet been proven to be effective antivirals or treatments against human noroviruses, these results indicated that an HMO cocktail of 2′FL-3FL might not work in a chronically infected patient with two distinct GII.4 variants. Clearly, additional studies with these HMOs as antivirals are needed.

In summary, our data indicated that the chronically infected patient produced numerous norovirus capsid quasi-sequences over the span of an infection. This is the first reported case of an individual cocontaminated with two GII.4 variants for such a long period. Similar capsid sequences are not yet reported in the database, suggesting that these viruses are not easily transmitted to other individuals. More importantly, since outbreaks with closely matching sequences have also not been reported, it could be that these viruses were less pathogenic than other GII.4 variants, although direct evidence is lacking.

## MATERIALS AND METHODS

### Chronic patient.

The male patient suffers from a complex X-linked syndrome and is under immunosuppressive therapy. During the study, the patient lived at home together with his parents and a healthy brother who was three years older. The patient was admitted to hospital within the study period only once for 5 days due to pneumonia. He had no severe diarrhea during the study period. He attended a school for disabled children, and no outbreaks of norovirus gastroenteritis occurred there within the 13-month study. In August 2009, the patient suffered from an acute norovirus infection that lasted approximately 6 days. Thereafter, norovirus could be isolated repeatedly, leaving him as a chronic norovirus carrier. However, the patient reported only a few symptoms of acute gastroenteritis that were directly norovirus associated during the past 6 years. In 2009, 2 days after the patient was hospitalized, his mother, who accompanied him in the hospital, suffered from an acute gastroenteritis infection that lasted 3 days. The visiting father also became ill with mild gastroenteritis for 2 days. At this time, neither parents’ stool was tested for norovirus. Subsequently, no further symptoms of gastroenteritis have occurred in the parents up to the present time. The elder brother had no reported cases of gastroenteritis. For this study, stool specimens were collected from the patient (July 2014 to July 2015; 13 months) and both parents (March 2015), with whom he resides. Additionally, sera from the patient and his parents, as well as saliva from the patient, were collected in July 2015. All materials were obtained after written informed consent and in agreement with the Declaration of Helsinki, federal guidelines, and the Local Ethics Committee (2015-589N-MA).

### Analysis of the capsid gene.

Initially, the partial capsid gene was amplified with broadly reactive primers using RT-PCR (44), and quasi-species were recognized in the sequence chromatograms (data not shown). Therefore, the region flanking the entire capsid gene (nt 5012 to 6956) was amplified using Phusion high-fidelity DNA polymerase (error rate, ~9.1 × 10^−6^ errors/bp/PCR cycle) (45) with gene-specific primers. RT-PCR products covering the RdRp-capsid genes (nt 3058 to 6956) were also determined in order to identify genetic recombination. The PCR products were cloned, and single colonies were sequenced (2). The viral load in each month was determined using quantitative RT-PCR (1). Genetic inference between closely related nucleotide sequences was performed by median-joining haplotype network analysis using PopART version 1.7. Homology models of P domain sequences that represented different genetic clusters were generated using two closely matching sequences (PDB identifiers [IDs] 4OOX and 5IYN) in order to display amino acid substitutions.

### Cross-reactivities of norovirus VLPs.

An antigen ELISA was used to determine the cross-reactivities of the norovirus VLPs with 11 different monoclonal antibodies raised against GII.4 strains and the patient’s and parents’ sera using an established method (42, 46). MaxiSorp 96-well microtiter plates were coated with 5 μg/ml VLPs. The plates were incubated for 1 h at 37°C, washed three times with phosphate-buffered saline (PBS)–0.05% Tween 20 (PBS-T), and blocked with 5% skim milk (SM) for 1 h at room temperature. After washing with PBS-T, 2-fold serially diluted antibodies in PBS-T-SM were added to the plates and incubated for an additional 1 h at 37°C. The plates were washed as before and reacted with horseradish peroxidase (HRP)-conjugated goat anti-mouse antibody for 1 h at 37°C. The plates were then developed with *o*-phenylenediamine (OPD) and H_2_O_2_ in the dark for 30 min at room temperature. Finally, the reaction was stopped with 3 N HCl, and absorbance was measured at an optical density of 490 nm (OD_490_). All experiments were performed in triplicate. The binding cutoff was set to an OD_490_ of 0.2, as previously determined for norovirus VLPs (31).

### PGM and saliva ELISA binding assay.

The binding of norovirus VLPs to porcine gastric mucin type III (PGM) and saliva was measured using a method previously described (24). Briefly, MaxiSorp 96-well plates were coated with 10 μg/ml PGM for 4 h at room temperature. The saliva samples (A- and B-type saliva) (24) were heated for 10 min at 95°C, briefly centrifuged, diluted 1:500 in PBS, and then used to coat plates overnight at 4°C. VLPs were 2-fold serially diluted, added to the plates, and incubated for 3 h at 18°C. The plates were washed and incubated with rabbit polyclonal GII.10 VLP antibody (cross-reactive with GII.4 VLPs) for 1.5 h at 18°C. Next, HRP-conjugated goat anti-rabbit polyclonal antibody was added to the plates and incubated overnight at 4°C. The plates were then developed as described above.

### Synthetic HBGA binding assay.

MaxiSorp 96-well plates were coated with 15 μg/ml VLPs overnight at 4°C, washed with PBS-T, and blocked with 5% SM. Synthetic HBGAs (A trisaccharide, B trisaccharide, H2 trisaccharide, LeA trisaccharide, and LeX trisaccharide; Glycotech) conjugated with phosphonoacetic acid (PAA)-biotin were dissolved in distilled water to 100 μg/ml. Twofold serial dilutions of synthetic HBGA were added to the plates and incubated for 2 h at 37°C. The plates were then washed, incubated with streptavidin-HRP for 1 h at 37°C, and processed as described above.

### HMO blocking assay.

Blocking assays were performed as described above, except that the VLPs (0.5 to 5 μg/ml) were pretreated with serially diluted HMOs (2′-fucosyllactose [2′FL] or 3FL) for 1 h at room temperature. PBS was used as a blank, and untreated VLPs were used as a binding reference for each plate. The OD_490_ of the untreated VLPs was set as the reference value corresponding to 100% binding. The percentage of inhibition was calculated as follows: [1 − (mean treated-VLP OD_490_/mean reference OD_490_)] × 100. The half-maximal inhibitory concentration (IC_50_) was determined using Prism software (version 6.0) (24).

### Accession number(s).

The nucleotide sequences determined in this study were deposited in GenBank under the following accession numbers: KX514170 to KX514370.
