# Expression of Concern for Doerflinger et al., “Human Norovirus Evolution in a Chronically Infected Host”

**DOI:** 10.1128/mSphere.00489-20

**Published:** 2020-06-10

**Authors:** American Society for Microbiology, Washington, DC, USA

## EXPRESSION OF CONCERN

The American Society for Microbiology (ASM) and *mSphere* are issuing this Expression of Concern to alert readers that there has been an authorship issue regarding the following publication:

Doerflinger SY, Weichert S, Koromyslova A, Chan M, Schwerk C, Adam R, Jennewein S, Hansman GS, Schroten H. 2017. Human norovirus evolution in a chronically infected host. mSphere 2:e00352-16 https://doi.org/10.1128/mSphere.00352-16.

*mSphere* has been notified by the German Cancer Research Center (DKFZ) that after an investigation by the DKFZ Commission for the Investigation of Scientific Misconduct it has been determined that Dr. Bishal Singh should be an author on the above-mentioned paper. Per the ASM Journals Ethics Portal on Authorship: “ASM will not adjudicate disputes about authorship. If a dispute occurs, we will refer the issue to relevant institutions.” ASM has requested an Author Correction by this article’s corresponding authors to reflect this change. This Expression of Concern is issued pending the Author Correction.

