# Supplementary figures and images for: Human Norovirus Evolution in a Chronically Infected Host
Source: mSphere. 2017 Mar 29;2(2):e00352-16. doi: 10.1128/mSphere.00352-16 (PMC5371696; doi:10.1128/mSphere.00352-16)

Figure S1

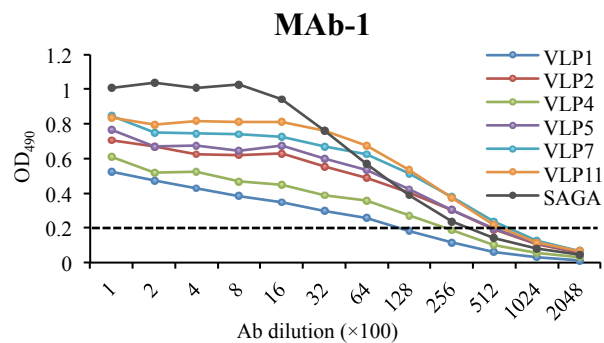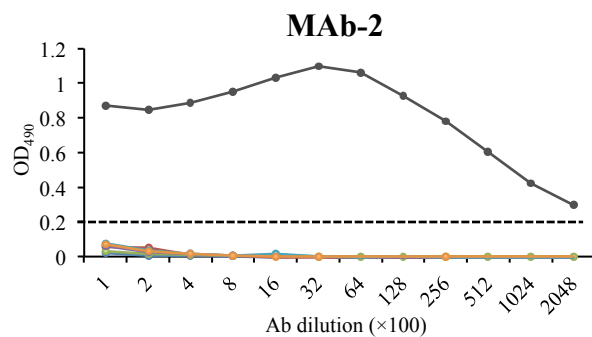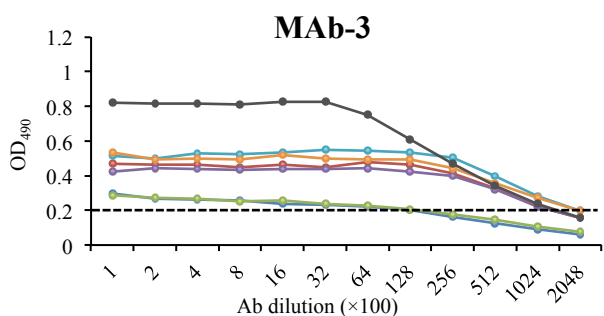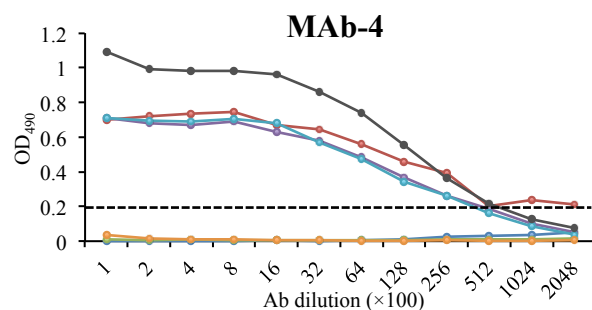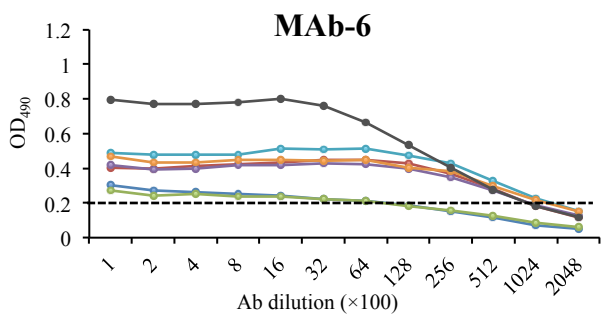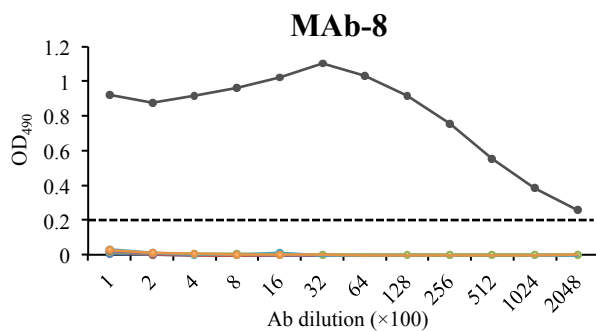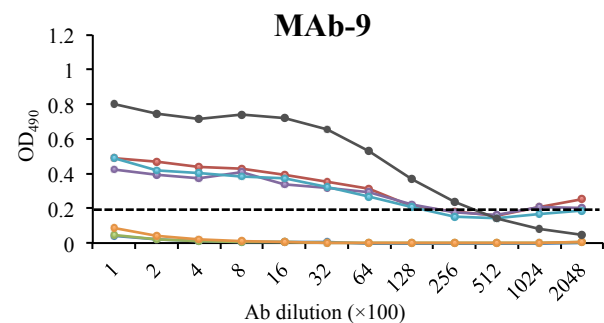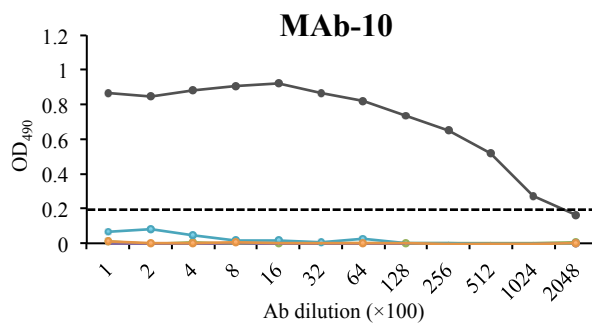

Supplement: FIG S1 [file sph002172257sf1.pdf]
